# Supplementary material for: The Role of the Respiratory Microbiome and Viral Presence in Lower Respiratory Tract Infection Severity in the First Five Years of Life
Source: Microorganisms. 2021 Jul 5;9(7):1446. doi: 10.3390/microorganisms9071446 (PMC8307314; doi:10.3390/microorganisms9071446)
Supplement: Supplementary file 1 [file microorganisms-09-01446-s001.zip › microorganisms-1261866-supplementary.pdf]

microorganisms

Supplementary Materials

Materials and Methods

Definitions of Outcomes

The primary outcome was the rate of hospitalization as a measure of disease severity. This was defined as admission to a hospital department other than the ED due to LRTI, irrespective of the duration of hospital stay. Secondary outcomes were strategy failure and the predicted risk of having bacterial LRTI. Strategy failure was defined by a predefined composite outcome and was based on disease course during the 7 days after ED presentation. The outcome consisted of secondary hospitalization during follow up, need for a switch in therapy (i.e. delayed antibiotic prescription, switch or adding of antibiotics) , oxygen dependency or fever up to day 7 or the development of complications, as defined in the original trial [1]. The predicted risk of having bacterial pneumonia was calculated using the Feverkidstool, a previously validated model to predict the presence of pneumonia or other serious bacterial infections in children with fever [1-3]. The Feverkidstool has been translated in a treatment decision model to guide antibiotic prescription in children with LRTI [1]. The model comprises of CRP (in mg/L) measurement and a range of clinical predictors, including age, sex, duration of fever in days, ill appearance, chest wall retractions, capillary refill time, hypoxia (oxygen saturation <94%), tachypnoea, tachycardia and temperature. A more detailed description of the Feverkidstool model has been published previously [1, 3]. Three separate risk groups were defined, with cut-offs set at <3% for low predicted risk for bacterial pneumonia, 4-10% for intermediate predicted risk and >10% for high predicted risk [1].

Data and Sample Collection

Clinical parameters assessment and CRP point of care testing (POCT) at ED presentation were performed by treating nurses and physicians. Data was collected clinically and from electronic health records. Collected data included general patient characteristics, presenting symptoms and physical signs, results of further investigations, management including antibiotic prescription and oxygen use, clinical parameters during contingent revisits and disease course. Follow-up data at seven days past initial presentation was collected through structured telephone calls or collected directly from patients and parents when children were hospitalized. CRP was obtained using Afinion AS 100 analyzers as POCT. Nasopharyngeal aspirates were obtained by the treating physician or nurse, using 0,9% NaCl, 1 ml per side. Samples were stored at -80 degrees Celsius.

Laboratory Procedures - Nanopore 16S rRNA Gene Sequencing and Classification of Bacteria

DNA extraction was performed using the AGOWA mag minikit (NAP40401, LGC Genomics, Berlin, Germany). The manufacturer's instructions were adjusted as follows; 200µl of nasal swab fluid, 200µl phenol and 150µl lysis buffer were added to the lysing matrix B (2ml tube, MP Biomedicals, CA, USA). As negative controls, 200 µL of lysis buffer was used instead of a sample. An additional bead beating step of 60 seconds at a power of 6m/s was performed using an MP Fast Prep-24 bead beater apparatus (MP Biomedicals, Irvine, CA, USA). Samples were centrifuged for 10 minutes (4000 rpm). 400 µL binding buffer and 10µl mag particles was added to 200 µL supernatant, vortexed, and the mix incubated for 2 min at room temperature. This was followed by incubation for 1 minute in a magnetic holder at room temperature. Supernatant was then removed and 130 µL wash buffer BL1 was added, followed by vortexing, incubation for 5 minutes at room temperature and a 1 min incubation in a magnetic holder. The supernatant was then removed and 70 µL wash buffer BL2 was added, vortexed and incubated for 5 minutes at room temperature, followed by a 1 min incubation in a magnetic holder. Again, the supernatant was removed, and the pellet centrifuged for 10 seconds at 10,000 rpm. The supernatant

was removed and 63 μL elution buffer was added. The manufacturer's instructions for the AGOWA mag minikit were then followed.

DNA concentrations were standardized to 10ng (a maximum of 10 μL could be added to a PCR mix) using the Quant-iT PicoGreen dsDNA Assay kit (P7589, Invitrogen, Carlsbad, CA, USA) followed by 16S rRNA gene sequence library preparation. The following procedures have been described previously by Heikema et al. [4]. 16S rRNA gene sequence libraries were prepared with the 16S Rapid Amplicon Barcoding Kit (Oxford Nanopore Technologies (ONT), SQK-RAB204) according to the standard procedures described by ONT. The complete 16S rRNA gene was amplified using 10 μL input DNA purified from nasal swabs, LongAmp Taq 2X master mix (New England Biolabs, Ipswich, MA) and the barcoded Nanopore sequence primers 27F 5′-AGA GTT TGA TCM TGG CTC AG-3′ and 149R 5′-CGG TTA CCT TGT TAC GAC TT-3′. DNA amplification was performed on a T100 Thermal Cycler (Biorad, Lunteren, the Netherlands) using the program; 1 min denaturation at 95˚C, 25 cycles (95 °C - 20s, 55 °C - 30s, 68 °C - 2 mins) and a final extension step of 5 mins at 65 ˚C. The 16S rRNA gene amplicons were quantified using Quant-IT™ PicoGreen™ (Thermo Fisher Scientific), equal amounts of amplicons per sample were pooled and the library was further processed as described by the manufacture. Next, the library was incubated with Library Loading Beads (ONT) and the mixture was added to the MinIon flow cell (ONT, R9.2). Sequencing was performed using a MinIon Nanopore sequencer (ONT) for approximately 16 hours.

Basecalling of sequence reads was performed using Guppy software (version 3.1.5+781ed57). Debarcoding and classification of bacteria was performed using EPI2ME (version 3.2.2) 16S workflow. Finally, a quality filtering step was performed was performed using a custom script with an average Q score set to 9 and identity of 85%.

Table S1. Additional baseline characteristics of the study population (n = 167).

General characteristics	N or median	% or IQR
Age groups		
Children 0–1 years	57	34.1
Children 1–2 years	47	28.1
Children 2–5 years	63	37.7
**Clinical presentation**		
Weight at ED visit in kg* (n = 165)	11	8.5–14.0
Respiratory Symptoms		
Cough (n = 163)	154	94.5
Dyspnea	133	79.6
Rales(n = 162)	91	56.2
Wheezing (n = 163)	54	33.1
Crackles (n = 163)	67	41.1
**Diagnosis**		
Probable cause of infection (n = 166)		
Probable bacterial	52	31.3
Probable viral	89	53.6
Unclear	25	15.1
**Investigations**		
Result Chest X-ray (n = 35)		
Normal	11	31.4
Focal infiltrate	18	51.4
Diffuse or perihilar abnormalities	6	17.1
**Risk factors and confounders**		
Mode of delivery (n = 143)		
Vaginal delivery	114	79.7
Caesarian section	29	20.3
Type of feeding (n = 140)		
Breastfeeding	84	60
Formula	38	27.1
Combination	18	12.9
Duration of breastfeeding (n = 86)		
< 3 months	31	36
> 3 months	55	64
Attendance of daycare (n = 139)	88	63.3
Presence of siblings under 5 (n = 141)	68	48.2
Frequency of RTI in first year of life (n = 140)		
0–2 times	99	70.7
3–4 times	21	15
5 times or more	20	14.3
Received AB at any time before ED visit (n = 137)	65	47.4
Number of AB courses received		
1 time	26	41.9
2–3 times	17	27.4
4 times or more	19	30.6
Received AB in 4 weeks before ED visit (n = 57)	16	28.1

Data are presented as total n and percentage or median with interquartile range indicated by *. If any data was missing, total n for that specific characteristic is reported. Definition of abbreviations: ED = emergency department, RTI = respiratory tract infection, AB = antibiotics.

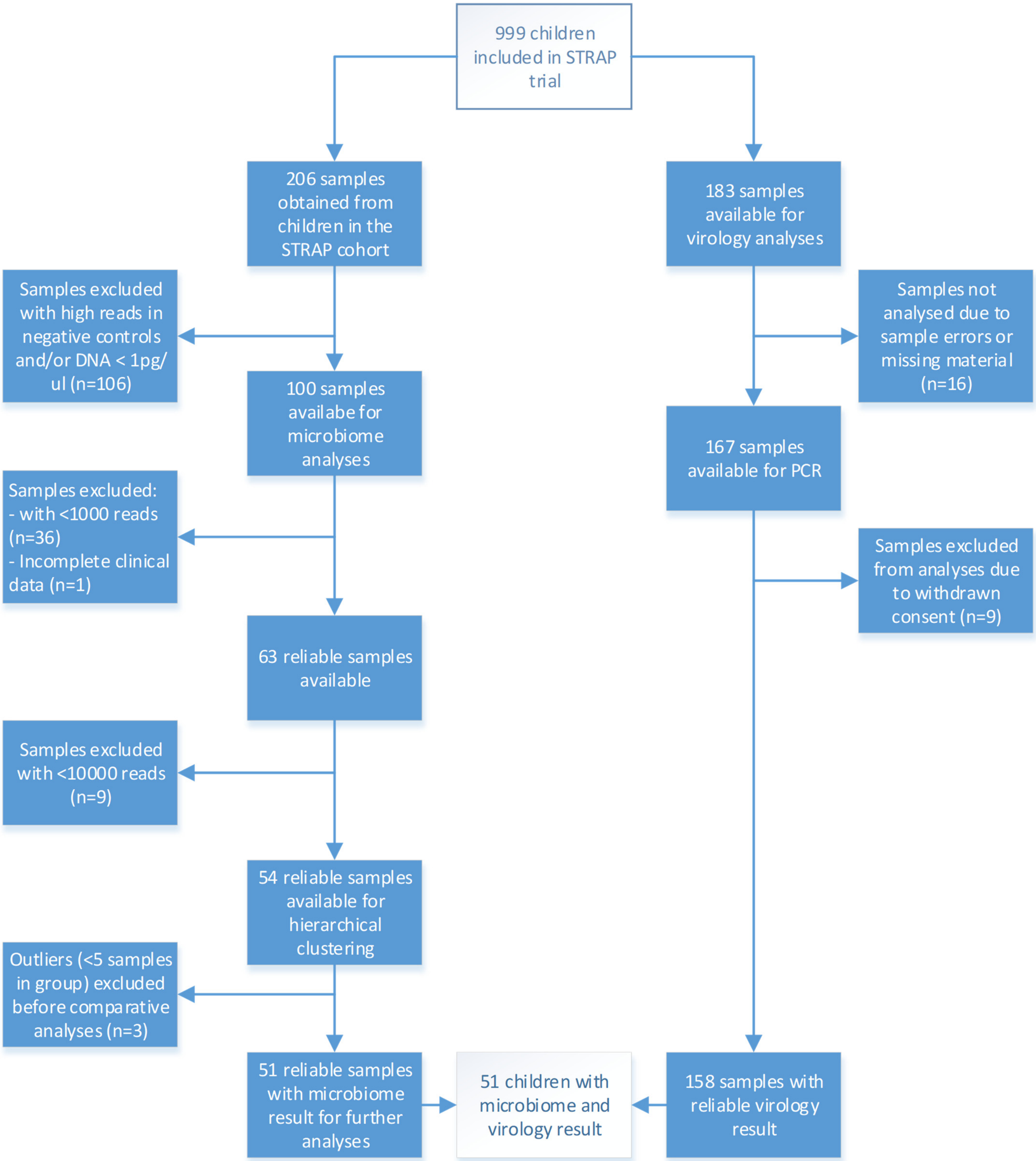

Figure S1. Flowchart of inclusion, quality measures and sample availability.

Table S2. Univariate analyses for outcome measures per viral virulence group (total n = 158).

Outcome	High virulence (n = 78)		Low virulence (n = 61)		Negative (n = 19)		*p*-value
	**n**	**%**	**n**	**%**	**n**	**%**	
Hospitalization	52	66.7	37	60.7	12	63.2	0.763
Strategy failure (n=152)	18	23.1	12	19.7	3	15.8	0.748
Predicted risk of bacterial LRTI[a] in %	15.0		10.0		9.0		0.307*
**Risk groups** (n = 137)							0.101
low	15	23.1	12	21.8	2	11.8	
medium	9	13.8	16	29.1	7	41.2	
high	41	63.1	27	49.1	8	47.1	

Data is presented as n and percentage for each virulence group. If any data was missing, the total n available for the outcome measure is reported in the first column. P-values were calculated by means of chi-squared test or Fisher's exact test, unless otherwise specified. [a] Predicted risk of bacterial LRTI was calculated with the Feverkidstool, the median risk is displayed. * *p*-value calculated with Kruskall-Wallis test. Definition of abbreviations: AB = antibiotics, LRTI = lower respiratory tract infection.

Table S3. Overall presence of genera/families (relative abundance, TSS).

**A *Genus* / Family**	**%**	**B Family**	**%**
Moraxella	27.79	Pasteurellaceae	39.8
Haemophilus	22.10	Moraxellaceae	28.0
Unclassified / Pasteurellaceae	17.76	Streptococcaceae	15.0
Streptococcus	15.07	Carnobacteriaceae	8.3
Unclassified / Carnobacteriaceae	8.12	Staphylococcaceae	2.6
Staphylococcus	2.59	Pseudomonadaceae	2.6
Pseudomonas	2.58	Burkholderiaceae	1.1
Burkholderia	1.03	Unclassified / Bacillales	0.5
Unclassified /Lactobacillales	0.45	Unclassified / Lactobacillales	0.4
Gemella	0.43	Corynebacteriaceae	0.4
Corynebacterium	0.38	Veillonellaceae	0.3
Veillonella	0.30	Neisseriaceae	0.3
Unclassified /Moraxellaceae	0.23	Peptoniphilaceae	0.2
Helcococcus	0.22	Unclassified / Bacilli	0.2
Neisseria	0.21	Unclassified / Gammaproteobacteria	0.1
Granulicatella	0.20	Prevotellaceae	0.1
Unclassified /*Bacilli*	0.15	Micrococcaceae	0.1
Unclassified,Gammaproteobacteria	0.11		
Prevotella	0.09		
Unclassified / Neisseriaceae	0.08		
Unclassified / Bacillales	0.05		
Psychrobacter	0.05		

Percent relative abundance of the top 20 genera (Table S2a) and their respective families (Table S2b) for all samples combined and after TSS normalization. For Table S2a - if taxa could not be assigned (Unclassified) at the Genus level, then Family level taxonomy is used. For Table S2b - if taxa could not be assigned (Unclassified) at the Family level, then order or Class level taxonomy is used.

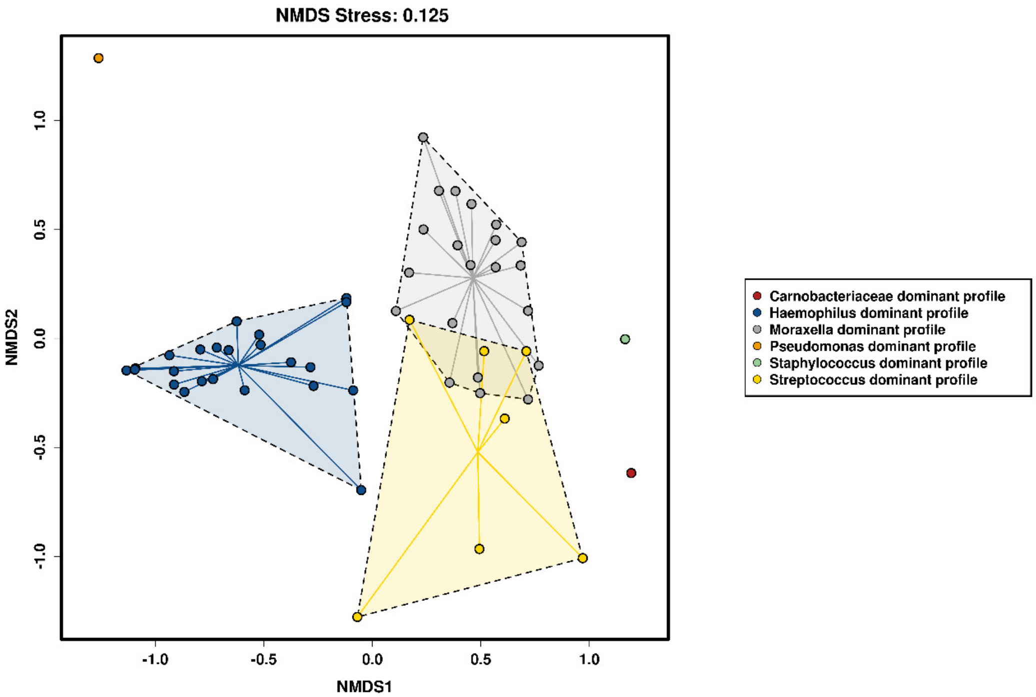

Figure S2. NMDS plot of microbiome profiles at genus level. Non-metric Multi-Dimensional Scaling (NMDS) plot of microbiome profiles at the genus level, visualizing multidimensional differences between profiles. Genus-dominant profiles for individual samples are presented by colorized dots.

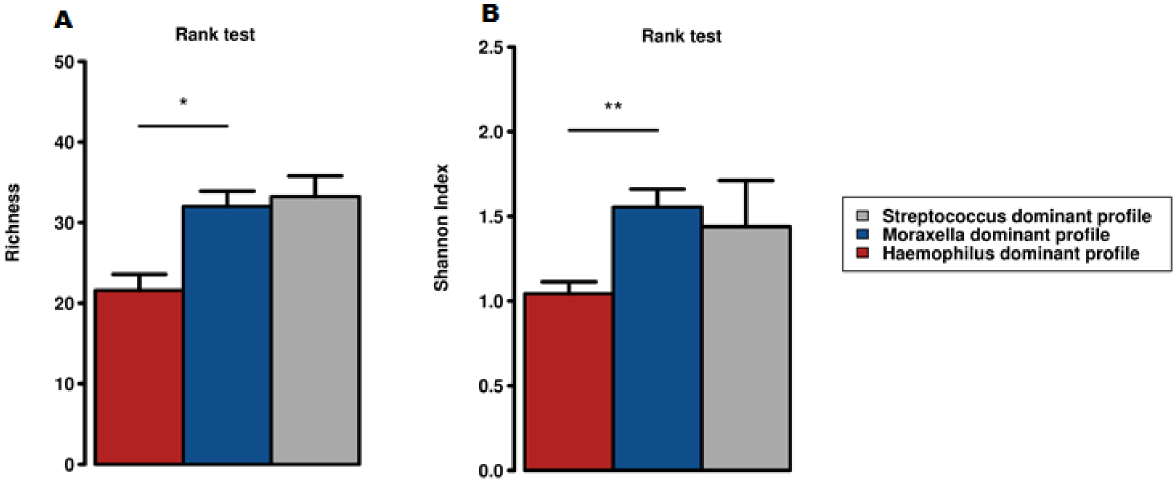

Figure S3. Richness & Alpha Diversity as measured by Shannon index. Diversity measures calculated at OTU level compared between genus-dominant microbiome profiles. p-values are calculated using the non-parametric Kruskall-Wallis test. *$p < 0.05$ ** $p < 0.01$. (**A**) Richness (**B**) Alpha diversity using the Shannon index.

Table S4. Univariate analyses for outcome measures per genus dominant microbiome profile (total n=51).

	Haemophilus	n = 23	*Moraxella*	n = 21	*Streptococcus*	n = 7	*p*-value
**Outcome**	**n**	**%**	**n**	**%**	**n**		**%**
Hospitalization	17	73.9	16	76.2	6	85.7	1
Strategy failure	5	21.7	2	9.5	1	14.3	0.656
Predicted risk of bacterial LRTI[a]	21.8		14.6		32.6		0.942*
**Risk groups**							
low	3	13.0	3	14.3	2	28.6	
medium	0	0.0	5	23.8	0	0.0	
high	15	65.2	11	52.4	3	42.9	

Data is presented as n and percentage for each microbiome profile. If any data was missing, the total n of the outcome measure is reported in the first column. [a] Predicted risk of bacterial LRTI was calculated with Feverkidstool. *p*-values were calculated by means of chi-squared test or Fisher's exact test for categorical outcomes. *p*-values for numerical outcomes were calculated by Kruskall-wallis test as indicated by *. Definition of abbreviations: LRTI = Lower respiratory tract infection.

Table S5. General characteristics and virology by genus dominant microbiome profile.

	Haemophilus	n = 23	*Moraxella*	n = 21	*Streptococcus*	n = 7	*p*-value
	**n**	**%**	**n**	**%**	**n**		**%**
Age (months)							
0–1 years	10	43.5	10	47.6	2	28.6	
1–2 years	8	34.8	7	33.3	1	14.3	
2–5 years	5	21.7	4	19.0	4	57.1	
**Gender**							
Male	15	65.2	16	76.2	3	42.9	
Female	8	34.8	5	23.8	4	57.1	
**Season**							
Spring	7	30.4	8	38.1	4	57.1	
Summer	0	0.0	4	19.0	0	0.0	
Autumn	5	21.7	6	28.6	2	28.6	
Winter	11	47.8	3	14.3	1	14.3	
**Virology**							
hMPV	5	21.7	3	14.3	2	28.6	0.704
influenza type A	0	0.0	0		0	0.0	
influenza type B	0	0.0	1	4.8	0	0.0	
parainfluenza	2	8.7	1	4.8	0	0.0	
RSV	6	26.1	6	28.6	2	28.6	1
adenovirus	6	26.1	2	9.5	0	0.0	
bocavirus	4	17.4	2	9.5	1	14.3	
enterovirus	1	4.3	2	9.5	0	0.0	
coronavirus	0	0.0	1	4.8	1	14.3	
rhinovirus	8	34.8	8	38.1	3	42.9	1
**Virulence groups**							0.697
High virulence	13	56.5	11	52.4	4	57.1	
Low virulence	5	21.7	6	28.6	2	28.6	
Negative	2	8.7	4	19.0	1	14.3	

Data are presented per microbiome profile as n and percentage. A *p*-value was calculated for viruses present in at least 10 samples and for the virulence groups, by chi-squared test. Definition of abbreviations: hMPV = human metapneumovirus, RSV = respiratory syncytial virus.

References

1. Van de Maat, J.S.; Peeters, D.; Nieboer, D.; van Wermeskerken, A.M.; Smit, F.J.; Noordzij, J.G.; Tramper-Stranders, G.; Driessen, G.J.; Obihara, C.C.; Punt, J.; et al. Evaluation of a clinical decision rule to guide antibiotic prescription in children with suspected lower respiratory tract infection in The Netherlands: A stepped-wedge cluster randomised trial. *PLoS Med.* **2020**, *17*, e1003034.
2. Nijman, R.G.; Vergouwe, Y.; Moll, H.A.; Smit, F.J.; Weerkamp, F.; Steyerberg, E.W.; van der Lei, J.; de Rijke, Y.B.; Oostenbrink, R. Validation of the Feverkidstool and procalcitonin for detecting serious bacterial infections in febrile children. *Pediatr Res.* **2018**, *83*, 466–476.
3. Nijman, R.G.; Vergouwe, Y.; Thompson, M.; van Veen, M.; van Meurs, A.H.; van der Lei, J.; Steyerberg, E.W.; Moll, H.A.; Oostenbrink, R. Clinical prediction model to aid emergency doctors managing febrile children at risk of serious bacterial infections: diagnostic study. *BMJ* **2013**, *346*, f1706.
4. Heikema, A.P.; Horst-Kreft, D.; Boers, S.A.; Jansen, R.; Hiltemann, S.D.; de Koning, W.; Kraaij, R.; de Ridder, M.A.; van Houten, C.B.; Bont, L.J.; et al. Comparison of Illumina versus Nanopore 16S rRNA Gene Sequencing of the Human Nasal Microbiota. *Genes* **2020**, *11*.
